# Dr. Davidson’s Case of Paracentesis Thoracis

**Published:** 1842-05-21

**Authors:** Wm. Davidson

**Affiliations:** Senior Physician to the Glasgow Infirmary


*Case of Paracentesis Thoracis.* By Wm. Davidson, M. D., Senior Physician to the Glasgow Infirmary.—The case detailed by Dr. Davidson is one of chronic pleurisy. Paracentesis was performed between the 6th and 7th ribs on the left side. Twenty pounds (apoth. weight) of serum were evacuated. It had a yellowish colour, was nearly transparent, adhesive, almost solidified by heat and nitric acid, and had a specific gravity of 1016. The fluid re-accumulated, and a small grooved needle was inserted into the left pleural cavity, near the cicatrix of the former opening, and, a large cupping-glass being applied over it, forty ounces of reddish-coloured serum were drawn off. Next day a small trocar was inserted by Dr. Lawrie, and twelve pounds (apoth. wt.) of serum were drawn off by cupping-glasses. The patient subsequently died.

Dr. Davidson makes some remarks on the mode of performing the operation. "The operation for empyema is not very frequently performed; for on the one hand there are the difficulties of the diagnosis, and even when the signs are pretty clear, there may be some doubt about the state of the lung; and on the other the danger of exciting a pleuritic inflammation. A plan, therefore, which may tend to remove or lesson any of these objections is worthy of consideration. The chief danger of the operation arises from the admission of air into the pleural cavity, and this cannot be avoided by the ordinary method, and if the stethoscope be applied to the chest during the evacuation of the fluid, atmospheric air can be heard entering its cavity with a noise similar to that produced by emptying a bottle nearly filled with water. To obviate this result, it occurred to me that the fluid might flow through the channel of a grooved needle, with the exhausting cupping-glass placed over it; this I accordingly tried, as stated in the report, and it succeeded perfectly; but the quantity of fluid being very great, it was necessary in the second trial, to use a small trocar. One practical difficulty occurred with the canula, but not with the grooved needle, viz., its liability to slip from the opening, when the cupping-glass was applied, which is obviated by previously tying it round the chest by a piece of narrow and very thin ribbon. The cupping-glass employed was curved, and capable of containing about two pounds of fluid, and it was exhausted by a piece of ignited lint, which had previously been dipped in alcohol. Very little air, as far as could be ascertained, entered the pleural cavity, and it is a good practical rule to hold the finger over the mouth of the canula, during the changing of the glasses, which ought to be frequently done, as it lessens the duration of the operation, by causing the fluid to flow in a full stream, whereas, without the aid of an exhausted cupping-glass, it would do so very slowly. The same plan of operation I have frequently adopted in opening chronic abscesses, even in the knee-joint, without any subsequent irritative fever or inflammation, and in one or two cases, even in cachectic constitutions, there has been no re-accumulation of matter, when firm bandaging was afterwards had recourse to."

In another case, the operation was conducted exactly as described in the former, and the narrow piece of ribbon employed was completely successful in retaining the canula. A few bubbles of air got admission into the pleura, which is inferred, as stated in the report, from the metallic tinkle, which was heard two or three times. This may, he thinks be obviated, by withdrawing the cupping-glass whenever the fluid ceases to flow in a stream, and applying another one.—*London and Edinburgh Jour. of Med. Science*, No. 11.

[Practically, the operation for empyema is almost abandoned. We do not conceive it to be justifiable, except where the oppression is so great that the patient is in imminent danger of suffocation. We do not mean to say that many patients do not recover from the operation, but that the chances of recovery are lessened by it, and the sufferings of the patients are in general increased. If we make the artificial opening, it is next to impossible to prevent the admission of air into the cavity of the pleura; this, of course, acts most deleteriously upon the surface of the abscess, and increases or developes the hectic fever; in other words, we make an artificial pneumothorax, with its necessary irritation. In the natural pneumothorax, nature makes an effort for the cure of the patient by producing a secondary empyema, and if the lesion is ever to be remedied, it is by the process of absorption of the purulent matter. It is true, that in the artificial pneumothorax, caused by paracentesis, we can, to a great extent, control the admission of the air, but we can rarely do so entirely; hence, we have almost always to regret the operation.

If it be thought absolutely necessary to puncture the chest, the operation as modified by Dr. Davidson seems to us to promise extremely well.]

